# Short Chain Fatty Acids Lower Inflammation and Restore Intestinal Integrity and Function Markers in *Mycobacterium paratuberculosis*—Infection In Vitro Model

**DOI:** 10.3390/nu17233663

**Published:** 2025-11-23

**Authors:** Piotr P. Lagod, Ahmad Qasem, Saleh A. Naser

**Affiliations:** Burnett School of Biomedical Sciences, College of Medicine, University of Central Florida, Orlando, FL 32827, USA; piotr.lagod@ucf.edu (P.P.L.); ahmad.qasem@ucf.edu (A.Q.)

**Keywords:** *Mycobacterium avium paratuberculosis*, MAP, Crohn’s disease, propionic acid, butyric acid, SCFAs, GI inflammation

## Abstract

**Background**: Infection with *Mycobacterium avium paratuberculosis* (MAP) is closely associated with Crohn’s disease (CD) development, where excessive inflammation and marked intestinal damage are observed. **Objectives:** In this study, the role of short chain fatty acids, including propionic acid (PPA) and butyric acid (BA), was evaluated in an in vitro model, mimicking CD characteristics. **Methods**: MAP-infected THP-1 macrophages were treated with 1 mM and 10 mM of PPA or BA, and the conditioned media was co-cultured in Caco-2 cells. **Results**: Both PPA and BA caused an M2 shift with significant downregulation (*p*-value < 0.0001) in pro-inflammatory markers at both the RNA and protein levels. The downregulation is most likely due to the antimicrobial properties of PPA and BA. MAP growth was inhibited by several folds in MGIT (Mycobacteria Growth Indicator Tube) culture media supplemented with PPA or BA. Dysfunctional Caco-2 intestinal epithelial cells’ integrity and function, due to MAP infection, were restored with PPA and BA treatment. Specifically, *NOX1* expression was significantly decreased in 10 mM of PPA or BA-treated cells (*p* < 0.001), as validated by RT-PCR and microscopy. PPA and BA restored tight junction integrity by decreasing *Claudin-2* expression in the MAP group. **Conclusions**: The data clearly demonstrated that short chain fatty acids contain anti-inflammatory and antimicrobial properties with downstream beneficial effects on damaged intestinal epithelial cells, suggesting potential benefits as a dietary supplement for CD patients, particularly those who are not pregnant, due to a possible increased risk of autism spectrum disorder (ASD) development in offspring associated with propionic acid exposure.

## 1. Introduction

Crohn’s disease (CD) is a chronic inflammatory bowel disease (IBD) with a complex etiology [1]. CD and ulcerative colitis (UC, which is also classified as an IBD) are on the rise in the United States, with 2.4 to 3.1 million affected patients [2,3]. Both CD and UC share chronic mucosal inflammation, but while UC is confined to the colon and rectum, CD can affect any part of the gastrointestinal tract (from the mouth to the anus). CD is also characterized by discontinuous inflammation (patches) scattered throughout the digestive tract with parts of healthy intestine located between the affected areas [4,5]. Additionally, the damage associated with CD can affect all layers of the intestine (transmural inflammation) [6].

The etiology of CD is complex and involves genetic and environmental factors, dysregulated immune response, and specific microbiota infection [7,8,9]. Infection with *Mycobacterium avium paratuberculosis* (MAP) is closely associated with CD development. MAP is the causative agent of Johne’s disease in ruminants [10,11], with strikingly similar symptoms to CD. MAP was also found to be present in the blood and breast milk of CD patients at much higher rates than healthy individuals [11,12].

The quality of life of patients suffering from CD is often significantly diminished [13,14]. Patients suffer from abdominal pain (often with cramping), diarrhea (often with the presence of blood), constipation, and nausea [15,16,17]. Strictures and fistulas are present in half of the CD patients within 10 years of diagnosis. CD patients have an increased risk of serious infection [18,19], as well as an increased risk of cancer development [20]. Besides symptoms involving the digestive tract, CD can also lead to fatigue, weight loss, anemia, joint pain, and malnutrition [21,22,23,24]. Malnutrition present in CD has several causes including reduced appetite (either as a result of nausea and abdominal discomfort or as a side effect of medication), malabsorption (due to intestinal damage and impaired epithelial transport), and rapid intestinal transit [22,25,26,27,28,29].

At the molecular level, CD is characterized by an increase in pro-inflammatory cytokines such as TNF-α, IL-6, and IL-12 [30,31]. It is also characterized by an increase in oxidative stress [32,33,34] and intestinal permeability [35,36]. An increase in intestinal permeability is often associated with increased levels of Claudin-2 (a pore-forming protein) [37,38]. One of the common therapies for CD consists of anti-TNF-α biologicals (Infliximab, Adalimumab, Certolizumab), which although are a safer and more targeted option than general immunosuppressants, still lead to side effects and interfere with the clearing of MAP infection [1,39,40]. Additionally, as many as 30% of patients do not respond to the therapy, and as many as 50% of initially responding patients will lose the clinical benefits from the treatment over time [41,42,43]. Thus, there is a great need to develop alternative therapies, especially for CD patients with MAP infection.

As thoroughly described in our earlier report, short chain fatty acids (SCFAs) include acetic (AA), propionic (PPA), and butyric acid (BA) [44]. They are byproducts of intestinal microbial metabolism, where indigestible fibers and resistant starches are fermented. AA accounts for 60%, PPA for 20%, and BA for 20% of the total SCFAs produced by microbial fermentation. SCFAs have potent antimicrobial and anti-inflammatory properties in the GI tract [45,46,47]. The anti-inflammatory activity of SCFAs is mediated mainly through G-protein coupled receptors (GPR41, GPR43, and GPR109A) and the inhibition of histone deacetylase, where neutrophils, monocytes, and macrophages alongside other cells are modulated [47]. Several studies showed that SCFAs are present at lower levels in IBD versus healthy populations [48,49,50].

Although SCFAs exhibit antimicrobial and anti-inflammatory effects, their efficacy is unknown in CD associated with MAP infection. In this study, an in vitro model of MAP infection associated with CD was utilized. THP-1 monocyte-derived macrophages were infected with MAP (UCF4 strain isolated from a patient with active CD), after which the conditioned media was transferred from THP-1 to a differentiated Caco-2 monolayer. The production of a pro-inflammatory cytokine (in THP-1 culture), oxidative stress, and tight junction integrity (in Caco-2 cells) was assessed, as well as the antimicrobial effect of PPA and BA on MAP in MGIT (Mycobacteria Growth Indicator Tube) media.

This study aims to establish proof of concept that SCFAs could be a potential dietary intervention for CD in patients with MAP infection, due to SCFAs’ anti-inflammatory and antimicrobial effects, especially against MAP. An increase in SCFAs could be accomplished by careful PPA and BA supplementation or by a change in the diet, where food high in fibers and resistant starches is introduced.

## 2. Materials and Methods

### 2.1. Cell Culture Maintenance and MAP Infection

THP-1 and Caco-2 cells were obtained from American Type Culture Collection (ATCC; Cat# TIB-202 and Cat# HTB-37, respectively, ATCC, Manassas, VA, USA). The THP-1 monocytes were cultured as suspension cells in a T75 flask (Cat# 12-565-349, Thermo Fisher Scientific, Nunc, Waltham, MA, USA) using RPMI-1640 (Cat# 30-2001, ATCC) media supplemented with 0.005 mM of 2-Mercaptoethanol (Cat# 31350010, Gibco, Thermo Fisher Scientific) and 10% fetal bovine serum (FBS; Cat# 30-2020, ATCC). The monocytes were counted and diluted to 5 × 10^5^ cell/mL, after which they were differentiated into macrophages by exposure to 50 ng/mL of phorbol 12-myristate 13-acetate (PMA; Cat# P8139-1MG, Sigma-Aldrich, St. Louis, MO, USA,) for 48h in a 12-well plate (Cat# 07-200-82, Corning, Corning, NY, USA). After differentiation, the media was completely removed in order to remove the PMA, and fresh media was supplied. In the pre-treatment group, differentiated THP-1 cells were exposed to a set concentration of PPA (propionic acid sodium salt; Cat# P5436-100G, Sigma-Aldrich) or BA (butyric acid sodium salt; Cat# 303410-5G, Sigma-Aldrich) dissolved in PBS (Phosphate-Buffered Saline; Cat# 10010023, Thermo Fisher Scientific) for 24h, after which the cells were infected with 2 × 10^4^ CFU of MAP per well. In the post-treatment group, the cells were infected with MAP 24h prior to the treatment with SCFAs. After the experiment concluded, the supernatant from the THP-1 cells used to treat the Caco-2 cells was stored at −80 °C for subsequent ELISA procedures.

Caco-2 cells were grown in a T75 flask in EMEM media (supplied with 20% FBS) as adherent cells, until they reached 80% confluency, upon which the cells were detached from the plate using Accutase (Cat# NC1793126, STEMCELL Technologies Vancouver, BC, Canada). Then, the cells were plated in 12-well plates (at a concentration of 2.5 × 10^5^ cell/mL) and allowed to be fully differentiated for 14 days. After complete differentiation, the cells were treated with PPA or BA at designated concentrations. For the experiment in which the Caco-2 cells were treated with supernatants from MAP-infected THP-1, 2/3 of the growth media from Caco-2 was removed and substituted with the same volume of media from THP-1 cells, upon which the cells were incubated for 24 h. At the conclusion of the experiment, the cells were lysed with lysis buffer (Cat# 9803, Cell Signaling, Danvers, MA, USA) for use in ELISA (as detailed in Section 2.4) or RNA was extracted utilizing the TRIzol method (as detailed in Section 2.2).

THP-1 cell viability upon MAP infection and PPA treatment was assessed. The cells were grown in suspension in a 12-well plate. The cells were infected with MAP and/or treated with PPA as described above. After a 48 h incubation, the cells were mixed with an equal amount of Trypan blue stain 0.4% (Cat#T10282, Invitrogen, Thermo Fisher Scientific), transferred to a Countess cell counting chamber slide (Cat# C10283, Invitrogen, Thermo Fisher Scientific), and counted using a Countess 3 FL automated cell counter (Invitrogen, Thermo Fisher Scientific). The cells highlighted in green by the instrument are considered live, while those highlighted in red are considered dead.

### 2.2. RNA Extraction and Gene Expression Quantification

The RNA was extracted using the TRIzol reagent (Cat# 15596026, Thermo Fisher Scientific), in accordance with the manufacturer’s protocol with minor modifications. Briefly, the cells were lysed with 1 mL per well (of a 12-well plate) of cold TRIzol, upon which the solution was transferred to a 1.5 mL microcentrifuge tube and 250 μL of Chloroform (Cat# C607-4, Fisher Scientific) was added, mixed thoroughly, and centrifuged at 10000 RCF (relative centrifugal force) for 5 min. The upper layer was carefully transferred to a clean 1.5 mL microcentrifuge tube, and 500 μL of ice-cold isopropyl alcohol (Cat# A461-4, Fisher Scientific) was added and mixed thoroughly. The solution was placed in −20 °C for 1 h, after which it was centrifuged at 13000 RCF for 45 min. The supernatant was removed, and the pellet was washed twice with 70% ethanol (ethanol (Cat# AC615090020) mixed with RNase free water (Cat# 10-977-015) to an obtained 70% *v*/*v* solution). After washing, the pellet was air dried and resuspended in 30 μL of DEPC-treated water (diethylpyrocarbonate; Cat# AM9906, Thermo Fisher Scientific). The RNA was quantified using NanoDrop One^C^ (Thermo Fisher Scientific). The cDNA was synthesized (following the manufacturer’s protocol) from 1000 ng of RNA per 20 μL reaction using High-Capacity cDNA Reverse Transcription Kit (Cat# 4368814, Applied Biosystems, Waltham, MA, USA). The cDNA was diluted with DNase/RNase free water, and gene expression was quantified using PowerUP SYBR Green Master Mix (Cat# A25742, Applied Biosystems) and QuantStudio 3 Real Time PCR System (Applied Biosystems). The primers used in the qPCR were obtained from Bio-Rad (Hercules, CA, USA) and had a proprietary sequence. The primers were as follows: GAPDH qHsaCED0038674; TNF-α qHsaCED0037461; NOX1 qHsaCED0041941. Gene expression was calculated using the 2^−ΔΔCt^ method.

### 2.3. Fluorescent Visualization of Protein and Oxidative Stress

Caco-2 cells were grown and dissociated as described in Section 2.1, after which they were plated in 8-chamber culture slides (Cat# 354108, Corning) at a concentration of 1 × 10^5^ cells per chamber. Then, the cells were allowed to differentiate for 14 days, after which the cells were treated with SCFAs or with supernatant from the THP-1 cells as described in Section 2.1. The cells were then fixed with 10% buffer formalin (Cat# 245-684) for 10 min, after which they were washed trice with PBS. The cells were permeabilized with 0.2% Triton X-100 (Cat#85111), washed trice with PBS, and blocked with 10% goat serum (GS; Cat#50062Z Invitrogen, Thermo Fisher Scientific). ZO-1 (zonula occludens-1) antibodies (Cat# MA3-39100-A488, Z01-1A12, Alexa Fluor 488, Invitrogen, Thermo Fisher Scientific) were diluted in 10% GS with the addition of 0.1% (*v*/*v*) Tween 20 (Cat#Bp337-100). Claudin-2 (Cat#710221, Recombinant Superclonal Antibody 3HCLC, Invitrogen, Thermo Fisher Scientific) and NOX1 (Cat# PA5-103220 Invitrogen, Thermo Fisher Scientific) were unconjugated; therefore, they required a secondary antibody (goat anti-rabbit IgG) conjugated to FITC (Cat# 65-6111, Invitrogen, Thermo Fisher Scientific). After incubation, the slides were washed five times with PBS and dried. VECTASHIELD Antifade Mounting Medium (Cat# H-1200-10, Vector Laboratories, Inc. Newark, CA, USA) containing DAPI (4′,6-diamidino-2-phenylindole) was added, and a cover slip was placed. The slides were imaged with an AmScope fluorescent microscope (AmScope IN480 Series Inverted Epi-fluorescence Trinocular Compound Microscope, with 6.0 Sony CCS sensor MF603C-CCD and 20X lens, AmScope Irvine, CA, USA) or a Zeiss axio M.2 microscope (Zeiss, Oberkochen, Germany). The pictures were edited with ImageJ software for MacOS (Version 1.53k, National Institutes of Health, Bethesda, MD, USA) and combined in Microsoft PowerPoint for MacOS (Version 16.100.4, Microsoft, Redmond, WA, USA). For the detection of reactive oxygen species (ROS) in Caco-2, DHE (dihydroethidium) was utilized. The cells were treated, fixated, permeabilized, and washed (as previously described), after which 100 μL of 1 μM DHE (Cat# D23107, Thermo Fisher Scientific) was added. After 30 min of incubation, the cells were washed trice with PBS, dried, and VECTASHIELD mounting media was applied. The cover slip was secured, and the slide was imaged with an AmScope fluorescent microscope (AmScope IN480 Series Inverted Epi-fluorescence Trinocular Compound Microscope, with 6.0 Sony CCS sensor MF603C-CCD and 20X lens). The pictures were edited with ImageJ software for MacOS (1.53k).

### 2.4. TNF-α Concentration Assessment

The concentration of TNF-α in the media supernatant was assessed using an ELISA kit (Cat# BMS223-4, Invitrogen, Thermo Fisher Scientific). Briefly, the supernatant from the THP-1 cells was stored in −80 °C. After thawing and mixing, 100 μL of supernatant was added to each of the wells in the ELISA kit plate, and all the wash and detection steps were carried out, in accordance with the manufacturer’s instructions. The detected absorbance was compared to a standard curve created based on the pure protein standard of known concentration supplied with the kit.

### 2.5. Measuring MAP Growth Inhibition by SCFAs

MAP clinical strain (UCF4) was propagated in modified Middlebrook 7H9 broth (supplemented with BD BACTEC MGIT ParaTB Supplement, Cat# 245156, Becton, Dickinson and Company, Franklin Lakes, NJ, USA; and Ferric Mycobactin J, Cat# 62-0002, Allied Monitor INC., Fayette, MO, USA) in the BACTEC MGIT 320 instrument (Becton, Dickinson and Company). The growth took place in tubes containing fluorescent growth indicator (BD BACTEC™ MGIT™ ParaTB Medium Tube, Cat# 245154, Becton, Dickinson and Company). The fluorescent compound was embedded in the silicone on the bottom of the tube. The fluorescence is quenched by the oxygen dissolved in the media. MAP growth depletes oxygen and allows for fluorescence to be detected by the instrument, which is converted to growth units (GU) based on the sets of standards. BA and PPA were dissolved directly in the media with supplements at 400 mM stock. Then, the stock was added to the tubes (after a specific volume of media was removed from the tubes to compensate for the addition of SCFA stock). 50 μL of active MAP culture was added to each tube supplemented with SCFAs, and MAP growth was recorded over time. At the conclusion of the experiment, the MGIT tubes with the fluorescent growth indicator were imaged with ChemiDoc. The resulting image was taken in a gray scale, after which it was colorized and edited with Image Lab software (version 6.1.0 build 7, Bio-Rad) to match the color of the indicator. Lastly, it was determined if the effect of the SCFAs on MAP at the end of the incubation period was bacteriostatic or bactericidal. At the end of the incubation period, the contents of the MGIT tubes were vortexed and placed in a 15 mL sterile conical tube (Cat# 339651, Thermo Fisher Scientific). Then, it was centrifuged for 10 min at 4500 RFC in an Eppendorf centrifuge (Cat# 5804R, Hamburg, Germany). The pellet was washed with fresh MGIT media, centrifuged, resuspended in new media (not containing SCFAs), and placed in a fresh MGIT tube. The tube was placed in a BACTEC 320 instrument and incubated for 25 days.

### 2.6. Measuring Non-MAP Antimicrobial Activity of SCFAs

Microorganisms used in this study included *Staphylococcus aureus* ATCC 29213, *Escherichia coli* ATCC 25922, and *Klebsiella pneumoniae* ATTC 13883. They were first grown on a TSA plate (Trypic Soy Agar plate; Cat# 22091-500G, Millipore Sigma, Burlington, MA, USA), after which the colonies were isolated, and the bacteria was grown overnight in TSB (Trypic Soy Broth; Cat# 22092-500G, Millipore Sigma). Then, the culture was diluted with fresh media before inoculation in a 96-well plate. Each well was supplemented with PPA or BA at a set concentration (in salt form or liquid unbuffered acid form). The plate was placed in a spectrophotometer (Agilent BioTek Synergy Neo2, Agilent, Santa Calara CA, USA) with double orbital shaking and at a temperature set to 37 °C. The optical density at 600 nm (OD600) was measured over time. The pH of the media was measured with Accumet AB315 (Fisherbrand, Cat# AB315, electrode Cat#13-620-223A, Fisher Scientific).

### 2.7. Statistical Analysis

All statistical analyses were performed using GraphPad Prism 10.4.1 software for MacOS (GraphPad Software, Boston, MA, USA). Normal distribution was checked with Shapiro–Wilk normality test. Significance was determined using an unpaired two-tailed t-test (for normally distributed data) and the Mann–Whitney U test (for non-normally distributed data). ANOVA with Dunnett’s multiple comparison test was used in experiments where more than two groups were present. A *p*-value lower than 0.05 (*), 0.01 (**), 0.001 (***), or 0.0001 (****) was considered significant.

## 3. Results

### 3.1. SCFA Treatment Did Not Significantly Affect the Viability of THP-1 Cells

**Figure 1:**
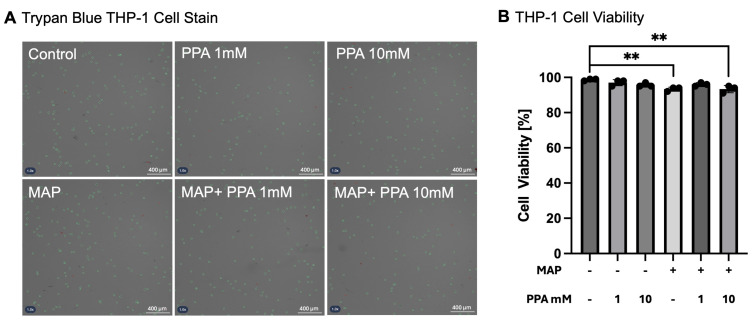
Viability of THP-1 macrophages upon PPA treatment and MAP infection. Subfigure (**A**) shows representative images from each group of treated cells. The green circles signify live cells, while the red circles represent dead cells. Subfigure (**B**) represents quantified cell viability, where live cells were compared to the total cells in each sample. Each group contains three biological replicates. The data is presented as mean ± SD with individual values marked on the graph. A *p*-value < 0.01 (**) is considered significant.

To test if PPA treatment of THP-1 cells affect their viability in a pure cell culture, the cells were exposed to 1 mM or 10 mM of PPA in either the presence or absence of MAP infection. Cell viability was measured with an automated cell counter and trypan blue stain. The cells highlighted in green in Figure 1A are considered live, while those stained red are dead. Cell viability was not significantly diminished by treatment with PPA alone but showed a slightly diminishing trend (Figure 1B), where the viability was 98.67% in the control cells (±0.58 pp) and 97.00% (±1.73 pp) and 95.67% (±1.15 pp) in 1 mM PPA and 10 mM PPA, respectively. In the MAP-infected cells without PPA treatment, the viability was 93.33% (±1.15 pp); thus, MAP infection reduced THP-1 viability by approximately 5%, confirming a mild cytopathic effect (*p* < 0.01). In the cells that were infected with MAP and simultaneously treated with 1 mM PPA, the viability was 96.00% (±1.00 pp), and 93.33% (±2.08 pp) in the 10 mM treated cells. This shows that treatment with PPA did not significantly affect the viability of THP-1 cells; however, MAP infection did significantly lower THP-1 viability (*p* < 0.01, *n* = 3).

### 3.2. SCFAs Downregulated TNF-α Pre-, At-, and Post-Infection with MAP

**Figure 2:**
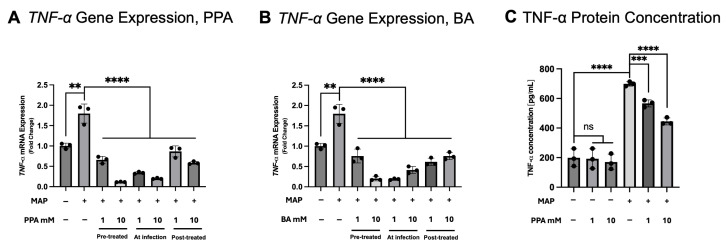
Gene expression and protein concentration of TNF-α in THP-1 macrophages in the presence of MAP infection and SCFA treatment. Subfigure (**A**) represents the gene expression of TNF-α (calculated with the 2^−ΔΔCt^ method) with MAP infection and PPA treatment. Subfigure (**B**) represents the gene expression of TNF-α with MAP infection and PPA treatment in THP-1 cells. Subfigure (**C**) represents protein concentration. Each group contains three biological replicates. The data is presented as mean ± SD with individual values marked on the graph. A *p*-value < 0.01 (**), *p* < 0.001 (***), and *p* < 0.0001 (****) are considered significant.

THP-1 monocyte-derived macrophages were infected with MAP and treated with 1 mM or 10 mM of PPA or BA. To better understand the best timing of PPA treatment, the THP-1 cells were treated in three distinctive timepoints. The cells were either pre-treated for 24 h prior to infection with MAP (labeled “Pre-treated” on the graphs in Figure 2), administrated simultaneously with MAP infection (labeled “At infection”), or 24 h after infection (labeled “Post-treated”). *TNF-α* gene expression was significantly increased by MAP infection (*p* < 0.01, *n* = 3) and consisted of a 1.793 (±0.24)-fold increase versus the control (Figure 2A). PPA treatment with either 1 mM or 10 mM concentration at all time points significantly lowered *TNF-α* gene expression (*p* < 0.0001, *n* = 3). When compared to the MAP-infected cells at the pre-treated time point, 1 mM and 10 mM PPA lowered *TNF-α* expression 0.367 (±0.050)-fold and 0.065 (±004)-fold, respectively. When PPA was administered simultaneously with MAP infection, *TNF-α* expression was 0.189 (±0.014)- and 0.111 (±0.008)-fold lower at 1 mM and 10 mM PPA versus MAP infection, respectively. At the post-infection time point, *TNF-α* expression was 0.482 (±0.075)- and 0.325 (±0.019)-fold lower at 1 mM and 10 mM PPA treatment versus MAP, respectively.

Treatment with BA showed similar efficacy, where all concentrations at all treatment times significantly lowered *TNF-α* expression (*p* < 0.0001, *n* = 3; Figure 2B). At pre-treatment, *TNF-α* expression was 0.421 (±0.095)- and 0.112 (±0.031)-fold lower than the MAP group in 1 mM and 10 mM BA treatment, respectively. When the cells were treated at the time of infection, *TNF-α* expression was 0.104 (±0.010)-fold lower at 1 mM BA and 0.230 (±0.047)-fold lower at 10 mM BA. At post-infection, the 1 mM BA treatment resulted in a 0.341 (±0.051)-fold decrease and the 10 mM treatment in a 0.421 (±0.050)-fold decrease.

At the protein levels, similar trends were present (Figure 2C). TNF-α protein concentration was significantly elevated in the MAP group versus the control, where in the control the concentration was 199.27 (±59.51) pg/mL and 698.79 (±15.12) pg/mL in the MAP group, which amounted to a 3.507-fold increase. The concentration of TNF-α in the MAP-infected cells with 1 mM PPA treatment was 566.89 (±23.73) pg/mL, which was a 0.811-fold decrease versus MAP infection. At 10 mM, the concentration was 444.90 (±23.71) pg/mL, which was a 0.637-fold decrease versus MAP infection.

### 3.3. SCFAs Significantly Lowered Oxidative Stress in the MAP-Infected THP-1-Caco-2 Culture Model

**Figure 3:**
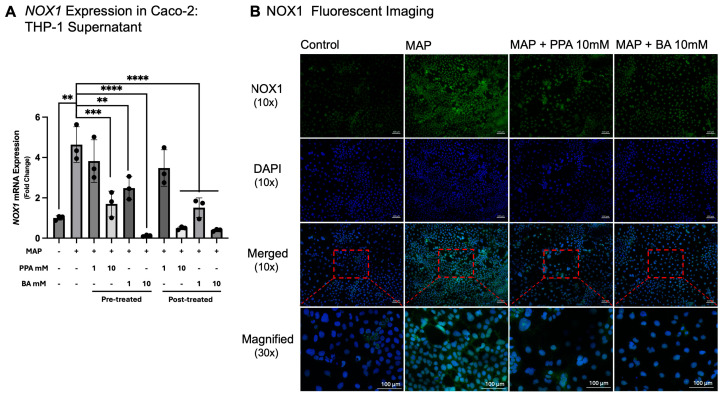
*NOX1* gene expression and NOX1 protein immunostaining in Caco-2 cells treated with SCFAs and THP-1 cell supernatants infected with MAP. Subfigure (**A**) represents *NOX1* gene expression (calculated with the 2^−ΔΔCt^ method). Subfigure (**B**) contains representative microcopy images of Caco-2 cells treated with SCFAs and supernatants from THP-1 infected with MAP. Green fluorescence signifies staining with anti-NOX1, while blue represents the nuclei of each cell (stained with DAPI). Images were acquired with 10× magnification. In the last row, the images were digitally magnified (area highlighted with a red rectangle) to better visualize the cells. Each group contains three biological replicates. The data is presented as mean ± SD with individual values marked on the graph. A *p*-value < 0.01 (**), *p* < 0.001 (***), and *p* < 0.0001 (****) are considered significant.

Supernatants from MAP-infected THP-1 cells were transferred to differentiated Caco-2 cells, upon which oxidative stress was assessed by measuring the gene expression of *NOX1* alongside fluorescent microscopy. *NOX1* expression was 4.62 (±0.88)-fold higher in the Caco-2 cells cultured with media from THP-1 cells infected with MAP versus the control where Caco-2 was cultured with media from THP-1 not infected with MAP (*p* < 0.01, *n* = 3; Figure 3A). When cells were pre-treated with PPA at 10 mM, *NOX1* expression was significantly downregulated (*p* < 0.001, *n* = 3, 0.365 ± 0.127-fold downregulation versus MAP). Cell pre-treatment with 1 mM BA and 10 mM BA also significantly downregulated *NOX1* gene expression (*p* < 0.01 and *p* < 0.0001 in 1 mM and 10 mM, respectively, *n* = 3; Figure 3A). The expression at 1 mM BA was 0.535 ± 0.122-fold lower than MAP, while with 10 mM BA treatment it was 0.026 ± 0.008-fold lower than MAP. At post-treatment with 10 mM PPA, 1 mM BA, and 10 mM BA, *NOX1* gene expression was also significantly downregulated (*p* < 0.0001, *n* = 3). In 10 mM PPA, the expression was 0.106 ± 0.016-fold lower than the MAP group, in 1 mM BA it was 0.326 ± 0.105-fold lower than the MAP group, and in 10 mM it was 0.085 ± 0.012-fold lower than the MAP group.

**Figure 4:**
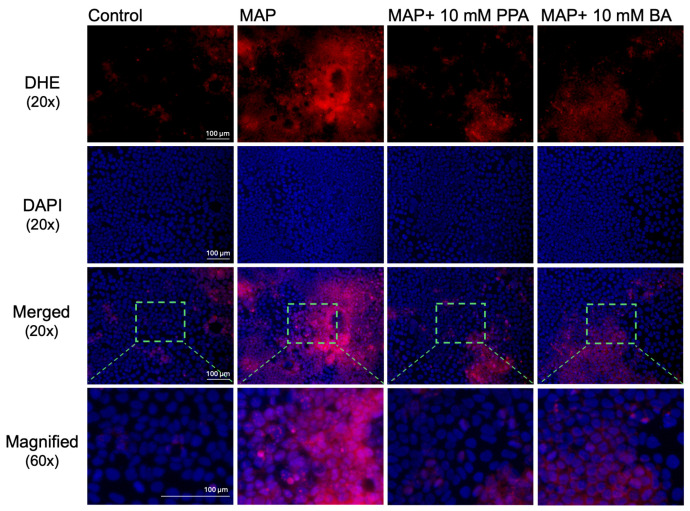
DHE staining of Caco-2 cells treated with SCFAs and THP-1 cell supernatants infected with MAP. Red fluorescence signifies staining with DHE, while blue represents the nuclei of each cell (stained with DAPI). The images were acquired with 20× magnification. In the last row, the images were digitally magnified (area highlighted with a green rectangle) to better visualize the cells.

Those findings were further validated with fluorescent immunostaining with anti-bodies against the NOX1 protein (Figure 3B). This was further supported with DHE staining (Figure 4), which detects ROS.

### 3.4. SCFA Treatment with PPA and BA Restored Tight Junction Integrity in the Caco-2 Monolayer

**Figure 5:**
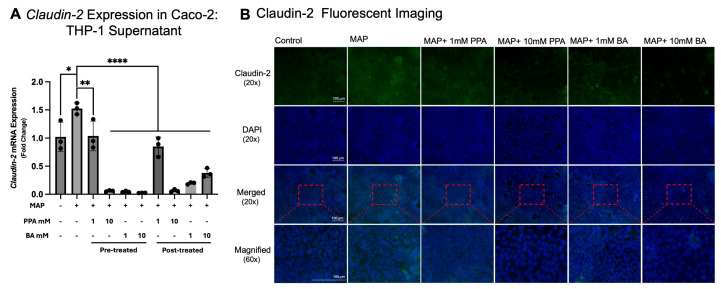
Claudin-2 gene expression and protein immunostaining in Caco-2 cells treated with SCFAs and THP-1 cell supernatants infected with MAP. Subfigure (**A**) represents *Claudin-2* gene expression (calculated with the 2^−ΔΔCt^ method). Subfigure (**B**) contains representative microcopy images of Caco-2 cells treated with SCFAs and supernatants from THP-1 infected with MAP. Green fluorescence signifies staining with anti-Claudin-2, while blue represents the nuclei of each cell (stained with DAPI). The images were acquired with 20× magnification. In the last row, the images were digitally magnified (area highlighted with a red rectangle) to better visualize the cells. Each group contains three biological replicates. The data is presented as mean ± SD with individual values marked on the graph. A *p*-value < 0.05 (*), *p* < 0.01 (**), and *p* < 0.0001 (****) are considered significant.

*Claudin-2* gene expression and fluorescent immunostaining were assessed to determine the effect of SCFAs on the integrity of tight junction in the Caco-2 CD in vitro model. Significant *Claudin-2* gene upregulation (1.469 ± 0.388-fold) was noted in the Caco-2 cells treated with supernatant from THP-1 cells infected with MAP versus the control (*p* < 0.05, *n* = 3; Figure 5A). Pre-treatment with 1 mM significantly (*p* < 0.01, *n* = 3) lowered *Claudin-2* expression versus the MAP group (0.680 ± 0.173-fold decrease). Pre-treatment with PPA at 10 mM concentration and BA at 1 mM and 10 mM also significantly lowered *Claudin-2* expression (*p* < 0.0001, *n* = 3), with a 0.039 ± 0.006-fold, 0.030 ± 0.008-fold, and 0.016 ± 0.001-fold decrease versus the MAP group, respectively. Post-treatment with SFCAs significantly (*p* < 0.0001, *n* = 3) lowered *Claudin-2* expression, with a 0.557 ± 0.111-fold decrease in 1 mM PPA, 0.042 ± 0.014-fold decrease in 10 mM PPA, 0.129 ± 0.012-fold decrease in 1 mM BA, and 0.248 ± 0.052-fold decrease in 10 mM BA.

**Figure 6:**
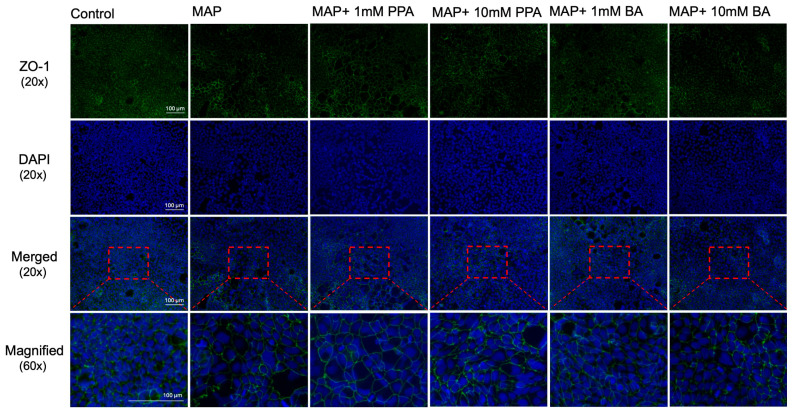
ZO-1 immunostaining microscopic image of Caco-2 cells treated with SCFAs and THP-1 cell supernatants infected with MAP. Green fluorescence signifies staining with anti-ZO-1, while blue represents the nuclei of each cell (stained with DAPI). The images were acquired with 20× magnification. In the last row, the images were digitally magnified (area highlighted with a red rectangle) to better visualize the cells.

Those findings were further validated by immunostaining with anti-Claudin-2, where representative images are included in Figure 5B. The Caco-2 cells were also stained with the ZO-1 tight junction protein (Figure 6). It was observed that the Caco-2 cells in the control showed a more uniform appearance and less areas on the slide where cells were absent versus MAP. PPA and BA treatment at 10 mM showed improvement in that trend; however, the monolayer did not appear as healthy as in the control.

**Figure 7:**
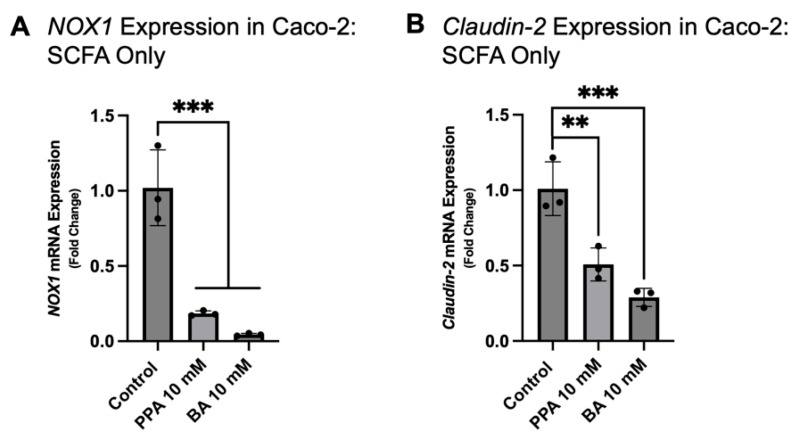
*NOX1* and *Claudin-2* gene expression in Caco-2 cells treated with SCFAs, without supernatants from MAP-infected THP-1 cells. Subfigure (**A**) represents the gene expression of *NOX1,* while Subfigure (**B**) represents the gene expression of *Claudin-2* (calculated with the 2^−ΔΔCt^ method). Each group contains three biological replicates. The data is presented as mean ± SD with individual values marked on the graph. A *p*-value < 0.01 (**) and *p* < 0.001 (***) are considered significant.

The effect of SCFAs on the Caco-2 pure cell culture (without the treatment with supernatants from THP-1 macrophages infected with MAP) was also assessed. *NOX1* gene expression was 0.181 ± 0.017-fold lower in 10 mM PPA treatment and 0.042 ± 0.008-fold lower in 10 mM BA versus the control (significant decrease, *p* < 0.001, *n* = 3; Figure 7A). *Claudin-2* gene expression was significantly decreased (*p* < 0.01, *n* = 3; Figure 7B) in 10 mM PPA treatment versus the control (0.502 ± 0.108-fold decrease). In 10 mM BA treatment, *Claudin-2* gene expression was also significantly decreased (*p* < 0.001, *n* = 3; Figure 7B) versus the control (0.287 ± 0.059-fold decrease).

### 3.5. SCFAs Inhibited MAP Metabolic Activity in MGIT Culture Media

**Figure 8:**
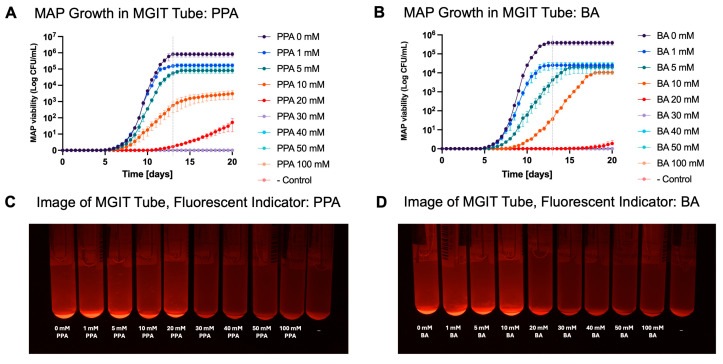
MAP growth over time in the presence of SCFAs. Subfigures (**A**,**B**) represent MAP growth in CFU/mL. The data is shown as mean ±SD on the logarithmic scale. PPA 0 and BA 0 represent the samples without any SCFAs added, while −Control represents the sample where no MAP was added to the MGIT tube. Subfigures (**C**,**D**) represent pictures of MGIT tubes where fluorescence signifies MAP growth.

MAP growth was inhibited by as little as 1 mM of PPA and BA. Figure 8A,B show the growth of MAP over a period of 20 days in the presence of PPA and BA, respectively. Growth is presented on the logarithmic scale. Table 1 shows the percent inhibition of MAP growth at 13 days (the time when the control group reached the stationary phase). 100% growth inhibition (both at 13 days and at the end of the experiment) was observed at 30 mM of PPA and 30 mM of BA. At the conclusion of the experiment (20 days), the MGIT tubes were visualized with a gel imager. MAP growth is indicated by fluorescence from the substrate that is activated when the oxygen in the media is lower due to consumption by actively growing bacteria. It was noted that at 30 mM and higher of PPA and BA, the fluorescence in the MGIT tubes was not present (Figure 8C,D). Also, at the conclusion of the experiment, the MGIT tubes where growth was not present were spun, and the pellet was washed and transferred to a new media tube. After the SCFAs were removed, MAP was able to resume growth, and it was determined that the effect of SCFAs on MAP is bacteriostatic (not bactericidal) below 100 mM.

### 3.6. SCFAs Inhibited Bacterial Growth in TSB Media

**Figure 9:**
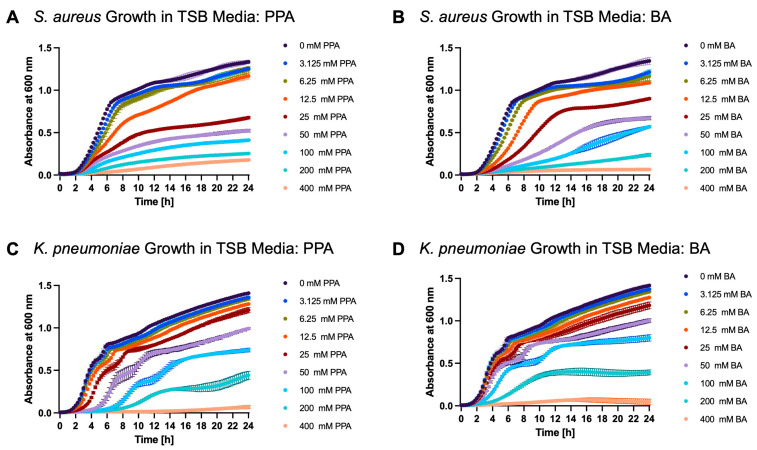
The growth curves of *S. aureus* and *K. pneumoniae* in the presence of PPA and BA. Each graph is shown as mean ±SD of absorbance at 600 nm (*n* = 3). Subfigures (**A**,**B**) show the growth of *S. aureus* in the presence of PPA and BA, respectively. Subfigures (**C**,**D**) show the growth of *K. pneumoniae* in the presence of PPA and BA, respectively.

*S. aureus, K. pneumoniae*, and *E. coli* were grown overnight in TSB media. Then, they were diluted and grown in the presence of SCFAs to determine their effect on bacterial growth. It was observed that *S. aureus* and *K. pneumoniae* growth was significantly diminished by both PPA and BA in a dose dependent manner. Figure 9A,B show the growth of *S. aureus* over time in the presence of various concentrations of PPA, while Figure 9C,D show *K. pneumoniae* in the presence of PPA and BA, respectively. Detailed in Table 2 are the percent inhibition values for PPA and BA for *S. aureus* and *K. pneumoniae* at 5 h, 10 h, and 15 h.

### 3.7. SCFAs Exerted Antimicrobial Effect Without Alteration of pH

Throughout this study, cell lines were treated with the sodium salts of propionic and butyric acid. However, the effect of unbuffered liquid (pure acid) form of those SCFAs on bacterial growth was also tested. PPA and BA were added at a concentration of 400 mM to TSB media or MGIT media, and the pH was measured. The addition of sodium propionate or sodium butyrate did not lower the pH of the TSB or MGIT media. However, the addition of unbuffered liquid propionic acid or butyric acid significantly lowered the pH (Table 3). While the pH of pure TSB media is 7.18, upon the addition of unbuffered liquid PPA, the pH dropped to 3.94. Upon the addition of unbuffered liquid BA, the pH dropped to 4.04. The same effect was seen in the MGIT media, where the pH of the media alone was 6.75 and dropped to 3.81 upon the addition of unbuffered liquid PPA or BA.

**Figure 10:**
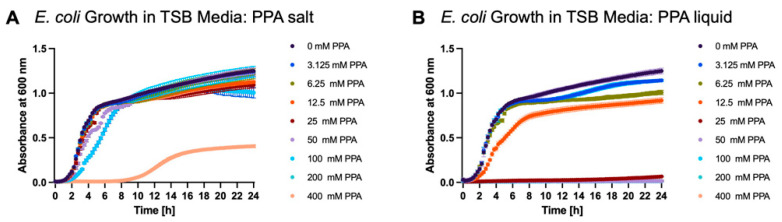
The growth curves of *E. coli* in the presence of PPA in sodium salt and unbuffered liquid (pure acid) form. Each graph is shown as mean ±SD of absorbance at 600 nm (*n* = 3). Subfigures (**A**,**B**) show the growth of *E. coli* in the presence of PPA in sodium salt and pure acid forms, respectively.

The effect of the addition of PPA as a sodium salt or unbuffered PPA was tested on *E. coli.* The bacterial culture was diluted from an overnight culture with fresh TSB, and 400 mM of PPA was added either in sodium salt form or unbuffered liquid. It was observed that the unbuffered liquid significantly lowered the pH of the media and had significantly higher antibacterial properties. In salt form, PPA only slightly inhibited the growth of *E. coli* until the concentration of 400 mM (Figure 10A). In liquid form, growth inhibition was much more potent (Figure 10B). Table 4 summarizes the growth inhibition of *E. coli* by PPA in two forms. For instance, at the 10 h time point, there was 5.1% *E. coli* growth inhibition at 25 mM of PPA in salt form, but at the same molarity, the growth inhibition of *E. coli* by unbuffered liquid PPA was 98.0%.

## 4. Discussion

Despite significant research efforts, CD is still considered incurable; thus, there is a great need to develop innovative treatments [3,51]. The burden of CD has both financial and personal factors. The yearly cost of a patient’s treatment ranges from $7824 to $41,829 when considering the additional loss of productivity due to CD symptoms [52,53]. Clinically, CD brings both intestinal and extraintestinal symptoms. The former includes pain, cramps, diarrhea, and the possible formation of strictures and fistulas, leading to necessary surgical procedures. The latter includes fatigue, weight loss, anemia, joint pain, and malnutrition. CD is also associated with psychological distress [52,54,55].

The current treatment for CD consists of anti-inflammatory drugs, immunosuppressants, antibiotics, and biologicals [56,57]. Although they show an effect on CD, they are burdened with side effects, especially with prolonged use [58]. Biologicals such as Infliximab, Adalimumab, and Certolizumab, which are one of the main CD treatments in up to 30% of patients, do not yield symptom improvement, and half of the patients who initially see symptom improvement stop responding over time [1,39,40,41,42,43]. Additionally, those treatments are very costly, especially if administered for a prolonged time. In CD associated with MAP infection, anti-inflammatory drugs and biologicals could worsen the infection, as they can prevent proper bacteria clearing by immune cells [1,39]. There is a significant body of studies attempting to use SCFAs in the treatment of IBD (both CD and UC). Most of the studies showed a reduction in inflammation upon SCFA treatment and overall symptom improvement, while several studies did not show any improvement. This could be caused by the heterogeneity in IBD’s etiology in the tested cases and diversity of the subjects. Additionally, those studies did not consider persistent infection with MAP [59,60,61].

This study aimed to provide proof of concept that supplementation with SCFAs either as a dietary intervention (consisting of food with a high content of fiber and resistant starches) or as careful PPA and BA supplementation can lead to improvement of symptoms in CD associated with MAP infection. This would offer a relatively inexpensive and safe alternative or supplemental treatment to the treatments that are currently offered. This study considers CD cases where their etiology involves infection with MAP, which is often overlooked in the treatment approach. Thus, the development of additional treatment options that can lower the excessive inflammation and target infection will provide the patient with dual and more effective therapeutic strategies. Beyond their well-documented anti-inflammatory activity, SCFAs exert direct antimicrobial and barrier-protective functions through multiple mechanisms. SCFAs such as butyrate and propionate disrupt bacterial membrane potential and inhibit enzymatic metabolism, leading to the growth suppression of both Gram-positive and Gram-negative pathogens [62,63]. These effects, combined with their capacity to enhance epithelial barrier function by tightening junctional complexes and restoring mucosal homeostasis [64], may explain the reversal of Claudin-2 and NOX1 upregulation observed in our model. In addition, SCFAs promote anti-oxidative signaling through the modulation of NADPH oxidase activity [65] and stimulate G-protein-coupled receptor pathways that attenuate TNF-α and IL-6 secretion [66]. Taken together, these findings support the notion that dietary or supplemental augmentation of SCFAs could represent a multifaceted therapeutic strategy for CD, addressing both microbial imbalance and epithelial dysfunction. This study utilized an in vitro model of CD with MAP infection that is well established by our laboratory [67,68].

In this study, it was first established that treatment with PPA and BA did not significantly alter the viability of the THP-1 monocyte-derived macrophages, and the changes in levels in the cytokines and another factor secreted by them is caused by downregulation of cytokines and not by lowering the number of live THP-1 cells. When the response to SCFA treatment was assessed in THP-1 cells, different time points were utilized to assess their effectiveness, either prior to MAP infection or when the infection had already occurred. Indeed, it was observed that the expression and protein levels of key pro-inflammatory cytokines seen in CD with MAP infection were significantly downregulated with both PPA and BA treatment; this was seen in all time points (when SCFAs were administered prior to, at the time, and post-infection). This shows that PPA and BA have potent anti-inflammatory effects on macrophages, which are one of the main mediators of inflammation in CD [69].

The supernatants from infected THP-1 cells were also added to the Caco-2 monolayer to mimic the damage of the intestine caused by extensive inflammation in CD [65]. There was a significant increase in *NOX1* (NADPH oxidase 1) expression in Caco-2 in the MAP group (more than four-fold), which is one of the main enzymes responsible for the production of ROS intestinal epithelia cells [70]. Upon both PPA and BA treatment, *NOX1* was significantly diminished versus the MAP group, especially at a 10 mM concentration, where it was close to the control. These findings were further confirmed with anti-NOX1 immunostaining and DHE staining that detects ROS.

Monolayer health and tight junction integrity was further evaluated. It was found that the treatment of Caco-2 with supernatants from MAP-infected THP-1 cells significantly increased *Claudin-2* expression, which was reversed by SCFA treatment. Claudin-2 is a pore-forming protein implicated in CD as one of the main “leaky gut” drivers [38]. This was further confirmed by microcopy with the anti-Claudin-2 fluorescent marker. Caco-2 cells were also stained with anti-ZO-1 antibodies to visualize cell borders. It was noticed that in the MAP group, there were more areas with no cells in the monolayer. This trend was reversed in 10 mM SCFA treatment. This showed that PPA and BA have the potential to reverse the damage to the epithelium caused by MAP infection in the in vitro CD model.

Finally, our data showed that PPA and BA not only have anti-inflammatory properties and can reduce damage to the epithelium but also have potent anti-MAP activity. 100% growth inhibition of MAP was observed in as little as 30 mM of PPA or BA, with significant growth decrease at even lower concentrations. The effect of PPA and BA was bacteriostatic, as removal of PPA from the growth media allowed for the re-establishment of MAP growth after 20 days. Additionally, it was shown that MAP is much more sensitive to SCFAs than other bacteria, which required a much higher concentration of PPA and BA to limit their growth. This further proves that PPA and BA are promising potential anti-MAP supplements. Our data also showed that the use of PPA and BA in sodium salt form does not alter the pH of the media. While the usage of PPA and BA in pure acid form lowers the pH and has even more potent antimicrobial properties, it may lead to over-acidification of the potential side effects.

This study provides proof of concept that PPA and BA could provide an alternative to other treatments for CD or potentially provide a supplementary treatment to boost the efficacy of biologicals. In this study, 1 mM and 10 mM of PPA or BA were chosen for the cell culture studies and 1 mM to 100 mM of either PPA or BA for the MAP bacterial cultures. PPA and BA were chosen as they are among the SCFAs to show the most potent anti-inflammatory properties (especially BA), and besides AA, they are the most abundant SCFAs [45,71]. The concentrations are physiologically relevant, as according to the literature, the concentration in the intestine is in the 20 mM range [72,73]. Interestingly, several studies showed that the concentration of SCFAs, especially BA, was significantly lowered in CD than in control subjects, in addition to a reduction in bacteria producing BA [49,74,75,76]. However, other SCFAs such as AA, valeric acid, and isobutyric acid should also be subjects of subsequent studies to better understand the roles of SCFAs in CD, as the human gut shows a large diversity of SCFAs. This study was limited to in vitro experimentation with only two cell types (THP-1 and Caco-2), which may limit the generalizability of the findings and conclusions. Future studies should include other immune cell types and possible mice models to better discern the effect of SCFAs in CD. The mice model could include a 2,4,6-trinitrobenzene sulfonic acid (TNBS)-model, where TNBS induces a Th1 immune response with transmural inflammation, ulceration, and diarrhea, which are hallmarks for CD [77,78]. This study focused on the overall effect of SCFAs on THP-1 cells and Caco-2 cells in terms of reducing inflammation and oxidative stress alongside increasing the integrity of the epithelial barrier. Futures studies should focus on the exact pathways affected by SCFA supplementation in the CD context. This could include the effect of SCFAs on G protein-coupled receptors (GPR41, GPR43, and GPR109A free fatty acid receptors) and their downstream pathways; these include PI3K-Akt, MEK-ERK, and p38 MAPK, which are involved in cell proliferation and inflammatory response [79,80]. Additionally, future studies should investigate the role of SCFAs as histone deacetylase inhibitors [81,82].

Additional research will be required to narrow down a dosage that would provide significant symptom reduction without evoking side effects, especially given that the usage of PPA during pregnancy could be linked to autism spectrum disorder (ASD) development [44,83,84]. BA might be a better candidate for supplementation in CD, as it was more effective than PPA and carries a lower risk of side effects associated with ASD [44,85]. Several clinical studies show that BA supplementation is relatively safe and does not evoke significant side effects [64,86,87,88]. Future studies should look at the combination of PPA and BA in the same treatment or the usage of SCFAs alongside other available treatments to improve their efficacy. The route of supplementation should also be considered. SCFAs could be increased through direct supplementation with PPA or BA via regular tablets, via targeted colonic delivery, or by increasing the intake of food rich in soluble fibers and resistant starches [89,90,91]. 

## 5. Conclusions

Overall, our data clearly shows that PPA and BA have potential as supplements in CD, especially in the presence of MAP infection, as they have both anti-inflammatory and anti-MAP activity.

## Figures and Tables

**Table 1:** Percent growth inhibition of MAP by PPA and BA in MGIT media.

		1 mM	5 mM	10 mM	20 mM	30 mM	40 mM	50 mM	100 mM
**13 days**	**PPA**	80.9% (±3.8 pp)	93.1% (±1.9 pp)	99.9% (±0.1 pp)	99.9% (±0.1 pp)	100% (±0.0 pp)	100% (±0.0 pp)	100% (±0.0 pp)	100% (±0.0 pp)
	**BA**	93.4% (±2.3 pp)	98.8% (±0.5 pp)	99.9% (±0.0 pp)	100% (±0.0 pp)	100% (±0.0 pp)	100% (±0.0 pp)	100% (±0.0 pp)	100% (±0.0 pp)

**Table 2:** Percent growth inhibition of *S. aureus* and *K. pneumoniae* by PPA and BA in TSB media.

mM ⟶		*S. aureus*								
		0	3.125	6.25	12.5	25	50	100	200	400
**5 h**	**PPA**	0.0%	14.1%	27.7%	39.6%	50%	57.4%	66.4%	75.6%	80.0%
	**BA**	0.0%	10.9%	30.7%	53.4%	66.4%	73.5%	77.5%	79.4%	81. 4%
**10 h**	**PPA**	0.0%	4.9%	9.7%	27.3%	47.9%	60.2%	68.2%	77.3%	84.6%
	**BA**	0.0%	2.2%	5.5%	12.9%	40.9%	66.9%	77.3%	83.0%	86.5%
**15 h**	**PPA**	0.0%	5.7%	6.6%	18.6%	46.2%	56.0%	64.4%	75.4%	82.1%
	**BA**	0.0%	6.2%	7.4%	12.9%	27.9%	50.7%	68.2%	80.8%	87.0%
**mM ⟶**		***K. pneumoniae***								
		**0**	**3.125**	**6.25**	**12.5**	**25**	**50**	**100**	**200**	**400**
**5 h**	**PPA**	0.0%	2.2%	4.8%	14.2%	33.2%	75.4%	82.3%	83.5%	84.5%
	**BA**	0.0%	1.2%	3.4%	7.5%	15.4%	25.3%	45.2%	75.0%	83.8%
**10 h**	**PPA**	0.0%	4.9%	7.5%	12.2%	17.9%	36.0%	57.6%	78.7%	86.6%
	**BA**	0.0%	3.0%	5.8%	10.6%	14.0%	19.8%	38.5%	56.8%	85.9%
**15 h**	**PPA**	0.0%	4.3%	6.6%	11.4%	19.9%	31.9%	44.4%	69.6%	90.1%
	**BA**	0.0%	2.7%	5.4%	11.2%	17.7%	27.7%	34.1%	60.1%	86.5%

**Table 3:** The pH of the TSB and MGIT media upon the addition of 400 mM of PPA or BA in sodium salt or unbuffered liquid (pure acid) form.

400 mM	TSB Media	MGIT Media
Pure media	pH: 7:18 (±0.01)	pH: 6.75 (±0.00)
Sodium Propionate Salt	pH: 7:18 (±0.01)	pH: 6.78 (±0.02)
Propionic acid (liquid)	pH: 3.94 (±0.01)	pH: 3.81(±0.01)
Sodium Butyrate Salt	pH: 7.21(±0.02)	pH: 6.84 (±0.01)
Butyric acid (liquid)	pH: 4.04 (±0.01)	pH: 3.81(±0.01)

**Table 4:** Percent growth inhibition of *E. coli* by PPA in sodium salt and pure acid form in TSB media.

mM ⟶		*E. coli*								
		0	3.125	6.25	12.5	25	50	100	200	400
**5 h**	**PPA Salt**	0.0%	1.4%	2.5%	1.8%	6.2%	25.3%	45.5%	76.0%	88.4%
	**PPA Liquid**	0.0%	1.24%	8.38%	41.6%	98.7%	99.4%	100%	100%	100%
**10 h**	**PPA Salt**	0.0%	1.8%	4.4%	6.2%	5.1%	5.1%	4.0%	35.4%	85.4%
	**PPA Liquid**	0.0%	7.5%	9.9%	22.0%	98.0%	100%	100%	100%	100%
**15 h**	**PPA Salt**	0.0%	0.28%	2.3%	7.2%	9.9%	1.1%	6.2%	29.7%	65.6%
	**PPA Liquid**	0.0%	10.6%	14.2%	16.4%	94.6%	98.9%	99.0%	100%	100%

## Data Availability

Raw data is available upon request.

## Abbreviations

The following abbreviations are used in this manuscript:

AA	Acetic acid
ASD	Autism spectrum disorder
ATCC	American Type Culture Collection
BA	Butyric acid
CD	Crohn’s disease
DAPI	4′,6-diamidino-2-phenylindole
DEPC	Diethylpyrocarbonate
DHE	Dihydroethidium
ELISA	Enzyme-linked immunosorbent assay
FBS	Fetal Bovine Serum
GAPDH	Glial fibrillary acidic protein
GI	Gastrointestinal
GPR	G protein-coupled receptor
GS	Goat serum
IBD	Inflammatory bowel disease
IL-6	Interleukin 6
MAP	*Mycobacterium avium paratuberculosis*
MGIT	Mycobacteria Growth Indicator Tube
NOX1	NADPH oxidase 1
PBS	Phosphate-Buffered Saline
PMA	Phorbol 12-myristate 13-acetate
PPA	Propionic acid
RCF	Relative centrifugal force
ROS	Reactive oxygen species
SCFA	Short chain fatty acid
SD	Standard deviation
TNBS	2,4,6-trinitrobenzene sulfonic acid
TNF-α	Tumor necrosis factor alpha
TSA	Trypic soy agar
TSB	Trypic soy broth
UC	Ulcerative colitis
ZO-1	Zonula occludens-1
